# Use of hybrid closed-loop insulin pump in pancreatogenic diabetes following necrotizing hemorrhagic pancreatitis: a case report

**DOI:** 10.3389/fcdhc.2025.1747454

**Published:** 2026-01-19

**Authors:** Laura Soldovieri, Gea Ciccarelli, Michela Brunetti, Gianfranco Di Giuseppe, Emanuele Gentile, Sara Sofia De Lucia, Antonio Gasbarrini, Alfredo Pontecorvi, Andrea Giaccari, Enrico Celestino Nista, Teresa Mezza

**Affiliations:** 1 Department of Translational Medicine and Surgery, Catholic University, Fondazione Policlinico Gemelli IRCCS, Rome, Italy; 2 Center for Endocrine and Metabolic Diseases, Fondazione Policlinico Gemelli IRCCS, Rome, Italy; 3 Pancreas Unit, Digestive Disease Center in the affiliation (CEMAD) Unit, Digestive Disease Center, Fondazione Policlinico Universitario A. Gemelli IRCCS, Rome, Italy

**Keywords:** case report, HCL system, insulin, pancreatitis, pancreatogenic diabetes

## Abstract

Pancreatogenic diabetes is a frequent and often underestimated consequence of acute and chronic pancreatitis. This form of diabetes shows clinical characteristics intermediate between type 2 and type 1 diabetes, presenting both insulin resistance and an insulin secretory defect that often requires earlier initiation of insulin therapy. We describe the case of a 60-year-old man with a history of obesity and family predisposition to diabetes who developed diabetes after necrotizing hemorrhagic acute pancreatitis complicated by portal vein thrombosis. The patient expressed great concern about his persistent hyperglycemia and marked glucose variability. Despite intensive treatment with oral antihyperglycemic agents and a basal-bolus insulin regimen, glycemic control remained suboptimal and characterized by wide fluctuations. Given the very high basal-to-bolus insulin ratio and the need for dynamic insulin delivery, the mylife Loop insulin delivery system was initiated. This resulted in a rapid and sustained improvement of glycemic control, with the Time in Range (TIR) increasing from 43% to 72% after one month and up to 88% at three months, while the Glucose Management Indicator (GMI) decreased from 7.9% to 6.5%. Benefits were stable at six months. To our knowledge, this is the first report describing the use of hybrid closed-loop insulin delivery system in diabetes following pancreatitis, and highlights how this technology can be particularly useful in achieving optimal glycemic control in patients in whom oral therapy is insufficient and conventional basal-bolus regimens are limited by the inability to personalize insulin delivery throughout the day. Automated insulin delivery proved effective in addressing the peculiar challenges of pancreatogenic diabetes, including high glycemic variability and a more complex management of prandial insulin therapy.

## Introduction

Pancreatogenic diabetes, also referred to as type 3c diabetes or diabetes of the exocrine pancreas, is a frequent yet underrecognized consequence of both acute and chronic pancreatitis. Its prevalence reaches approximately 30% among individuals with a history of pancreatitis (1) and is associated with increased cardiovascular and pancreatic cancer risk (2). Endocrine dysfunction may develop following acute or chronic inflammatory injury to the pancreas, particularly in individuals with pre-existing metabolic risk factors such as obesity or hyperlipidemia (3, 4). In necrotizing forms of pancreatitis, the risk of diabetes is notably higher (5).

Although pathophysiological mechanisms may partially overlap between post-pancreatitis diabetes and type 2 diabetes (6), the inflammatory and fibrotic pancreatic milieu in post-pancreatitis diabetes—depending on disease severity—leads to a more rapid and profound dysfunction of pancreatic islets. Neural injury further compromises endocrine regulation by reducing pancreatic polypeptide secretion (7). In addition, exocrine pancreatic insufficiency not only delays nutrient digestion and absorption but also impairs the secretion of gut-derived hormones, thereby disrupting the incretin–insulin axis. As a consequence, post-pancreatitis diabetes is more challenging to manage than type 2 diabetes: despite comparable HbA1c levels, patients experience greater glycemic variability and a higher risk of hypoglycemia, driven by malabsorption and impaired glucagon responses (8, 9). These distinctive pathophysiological features may be more effectively addressed by automated insulin delivery (AID) systems than by standard insulin therapy.

This case is of particular interest because it offers a novel therapeutic approach to a complex case of pancreatogenic diabetes. The patient’s clinical background, characterized by strong predisposing factors for type 2 diabetes and the development of persistent hyperglycemia after a single episode of necrotizing pancreatitis, made the diagnosis and management particularly challenging. The strategy combining oral antihyperglycemic agents and insulin proved insufficient to achieve adequate metabolic control. The introduction of mylife Loop provided an effective solution, enabling personalized and dynamic insulin delivery adapted to daily variations in insulin needs — a crucial aspect in patients with unstable glycemic profiles.

The novelty of this case lies in the successful use of a HCL insulin delivery system in diabetes following pancreatitis, namely the mylife YpsoPump combined with the CamAPS algorithm. While AID systems are widely validated in type 1 diabetes (10–12), their use in diabetes secondary to pancreatic disease remains exceptional. Notably, in the case series by Touimer et al. (13), advanced hybrid closed-loop systems -not including mylife Loop- were used in five patients with pancreatitis-related diabetes, whereas in the randomized controlled trial by Boughton et al. (14), a fully closed-loop system was employed in three individuals with this condition.

This experience demonstrates that such technology can overcome the limitations of conventional basal-bolus therapy, offering a more physiologic and adaptable insulin replacement. With the growing diffusion of AID systems, their application may increasingly extend beyond type 1 diabetes, representing a promising option for complex and mixed-pathophysiology forms such as pancreatogenic diabetes.

## Case description

A 59-year-old man was admitted to the emergency department for an episode of necrotizing hemorrhagic acute pancreatitis caused by gallstone disease (lithiasis), complicated by portal vein thrombosis and acute respiratory failure associated with bilateral basal pleural effusion in September 2022. At that time, he had no prior history of diabetes, but he already had established risk factors for diabetes: he weighted 120 kg with a height of 180 cm, corresponding to a BMI indicative of obesity (37 kg/m^2^), he suffered from hypertension and obstructive sleep apnea syndrome and he had a family history of diabetes (his grandfather was affected). During hospitalization for pancreatitis, he experienced persistent hyperglycemia, requiring basal-bolus insulin therapy, and lost approximately 20 kilograms. At discharge, this insulin regimen was maintained as follow: insulin glargine 18 UI, insulin aspart 6UI before breakfast, 8UI before lunch and 6 UI before dinner. The patient also developed exocrine pancreatic insufficiency and therefore initiated pancreatic enzyme replacement therapy (PERT), which was gradually titrated up to 70,000 UI of pancrelipases per meal. He also continued his hypertensive treatment with telmisartan 80 mg daily and bisoprolol 2.5 mg daily and started a therapy with ursodessosicolic acid 300 mg three times per day. Prior to this event, the patient had been hospitalized once for right hernioplasty surgery in 2018.

Beginning of 2023, CT imaging revealed a Walled-off Pancreatic Necrosis (WOPN). The patient was admitted to our Hospital in March 2023, for endoscopic drainage of a pancreatic collection. Subsequently, the peripancreatic collection became infected, and the patient was hospitalized for endoscopic necrosectomy three times between March and May 2023. At this time, patient’s weight was 88 kg (BMI 25.7 kg/m^2^) and probably due to this weight loss, therapy with telmisartan was suspended for blood pressure normalization, while bisoprolol continued.

Following these hospitalizations, the patient was referred to our center for further management. He was evaluated and managed by both the gastroenterologists of our University Hospital’s Pancreas Unit and the diabetologists of the Center for Endocrine and Metabolic Diseases.

### Therapeutic intervention

Starting from the first evaluation in July 2023, and during subsequent follow-up visits, the therapeutic regimen for diabetes was progressively optimized. Continuous glucose monitoring (CGM, Freestyle Libre 2) was initiated. Rapid-acting insulin was gradually tapered, while basal insulin was maintained (insulin glargine U300 raised up to 40 units daily) and oral antihyperglycemic agents were introduced: an SGLT2 inhibitor (dapagliflozin 10 mg daily), a DPP-4 inhibitor (alogliptin 25 mg daily), pioglitazone 30 mg daily, and metformin 2000 mg twice daily, the latter introduced with gradual titration.

Indeed, during hospitalization, an insulin regimen is the standard and preferred approach for inpatient management, representing the most effective strategy to control the initial glycemic decompensation. However, once the acute event has resolved, alternative therapeutic options can be considered. Clinical trials evaluating the efficacy and safety of various antihyperglycemic drugs in DEP are lacking, and therapeutic decisions should therefore rely on clinical judgment. Our patient presented with several risk factors for type 2 diabetes, which supported the choice of a combined regimen. Metformin was selected for its insulin-sensitizing and glucose-lowering effects, as well as its reported antineoplastic properties (15). Multiple meta-analyses have confirmed that DPP-4 inhibitors are not associated with an increased risk of pancreatitis (16, 17); therefore, a DPP-4 inhibitor was introduced to address the disruption of the incretin–insulin axis. The SGLT2 inhibitor was added with caution, in the context of continued basal insulin therapy and close monitoring.

Despite this multidrug regimen, glycemic control remained suboptimal at the 6-month follow-up (January 2024), prompting reintroduction of prandial insulin: HbA1c 67 mmol/mol; CGM metrics of the last 30 days: TIR (Time in range 70–180 mg/dl) 29%, TAR1 (Time above range 181–250 mg/dl) 42%, TAR2 (Time Above Range >250 mg/dl) 27%, TBR1 (Time below range 54–69 mg/dl) 2%, TBR2 (Time below range <54 mg/dl) 0%. The patient, therefore, continued on oral antihyperglycemic therapy combined with basal insulin glargine U300 (40 units daily) and 12–15 units of rapid-acting insulin per day.

Over the following months, he required several additional hospitalizations for endoscopic drainage of walled-off necrosis and repeated necrosectomies due to superimposed infection. During this period, he experienced significant weight loss, reaching 83 kg by 2025 (BMI 25.6 kg/m²).

Despite full adherence to therapy and a structured diet with fractional carbohydrate intake over five meals daily, glycemic targets were not achieved. (October 2024: HbA1c 61 mmol/mol; CGM metrics of the last 30 days: TIR 58%, TAR1 29%, TAR2 12%, TBR1 1%, TBR2 0%). During 2024, the patient was trained in carbohydrate counting and began to calculate mealtime insulin doses using an insulin-to-carbohydrate ratio of 1:40 and an insulin sensitivity factor of 1:50. However, this approach did not yield a substantial improvement in glycemic control (December 2024: HbA1c 63 mmol/mol; CGM metrics of the last 30 days: TIR 43%, TAR1 40%, TAR2 16%, TBR1 1%, TBR2 0%).

Given the marked glycemic variability and the need for refined modulation of insulin delivery across the day, after multidisciplinary discussion and shared decision-making with the patient, in February 2025 he was transitioned to a hybrid closed-loop (HCL) system to improve glycemic control and minimize glucose fluctuations. Specifically, we chose the mylife YpsoPump combined with the Freestyle Libre 3 Plus CGM sensor with the mylife CamAPS FX algorithm for two main reasons: its highly customizable settings, including the possibility to choose up to 48 different glucose targets throughout the day, and our belief that it represented the most appropriate system for meal management in this specific case. Indeed, beyond its specific feature of slow-digestion meal, the system can increase basal insulin delivery during the post-prandial phase, which we consider the optimal strategy to manage hyperglycemia when digestion is delayed, as administering a larger meal bolus could otherwise lead to post-prandial hypoglycemia. Initial pump settings included an insulin-to-carbohydrate ratio of 1:45 for breakfast and dinner and 1:35 for lunch, an insulin sensitivity factor of 1:50, and a target glucose level of 120 mg/dL throughout the day. Upon initiation of HCL therapy, SGLT2 inhibitor was discontinued, while DPP-4 inhibitor, metformin, and pioglitazone were maintained.

### Follow-up and outcomes

**Figure 1:**
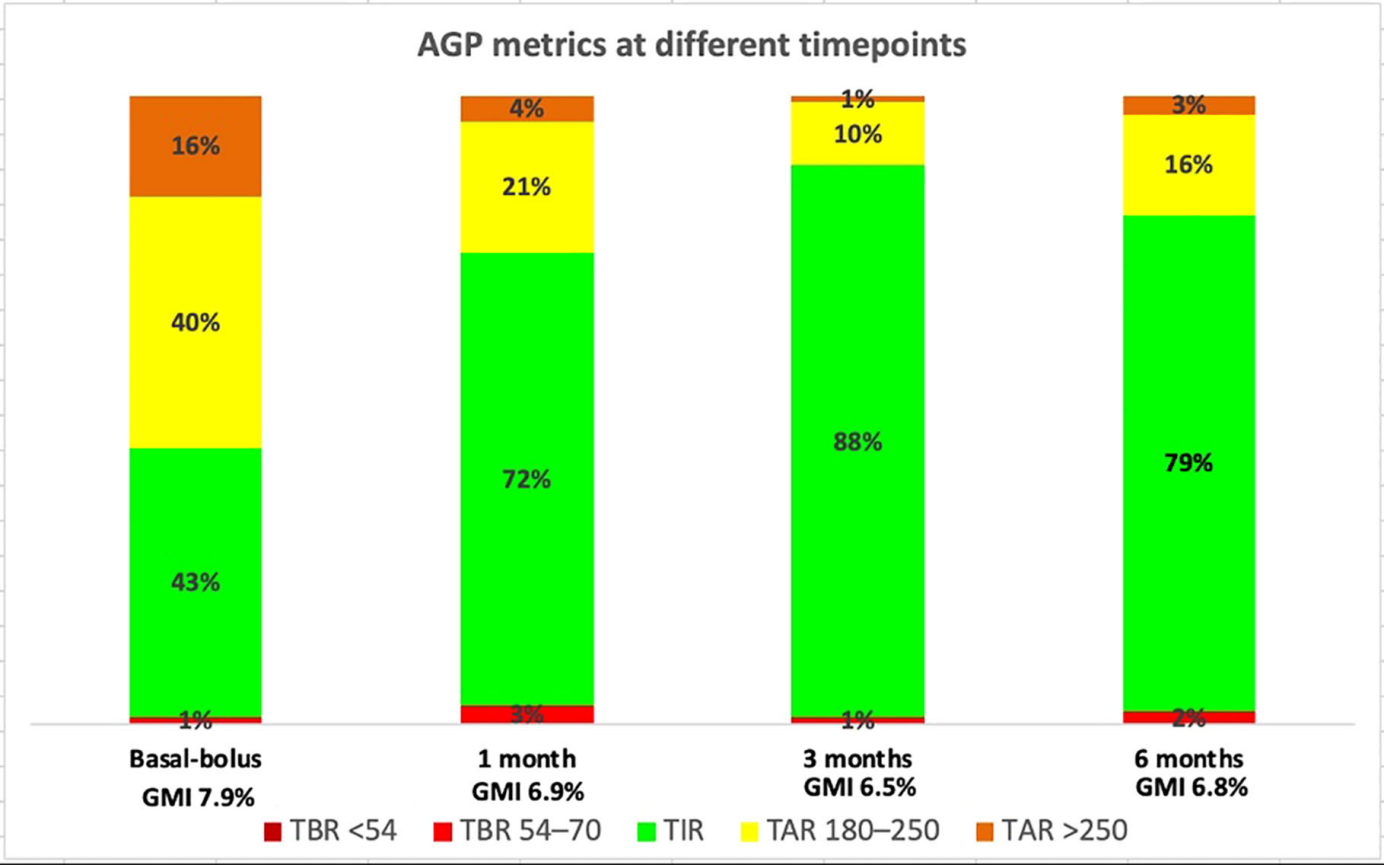
Stacked bar charts showing the percentage of time spent in different glucose ranges (TBR2 <54 mg/dL, TBR1 54–70 mg/dL, TIR 70–180 mg/dL, TAR1 180–250 mg/dL, and TAR2 >250 mg/dL) during basal-bolus therapy and at 1, 3, and 6 months after intervention.

**Figure 2:**
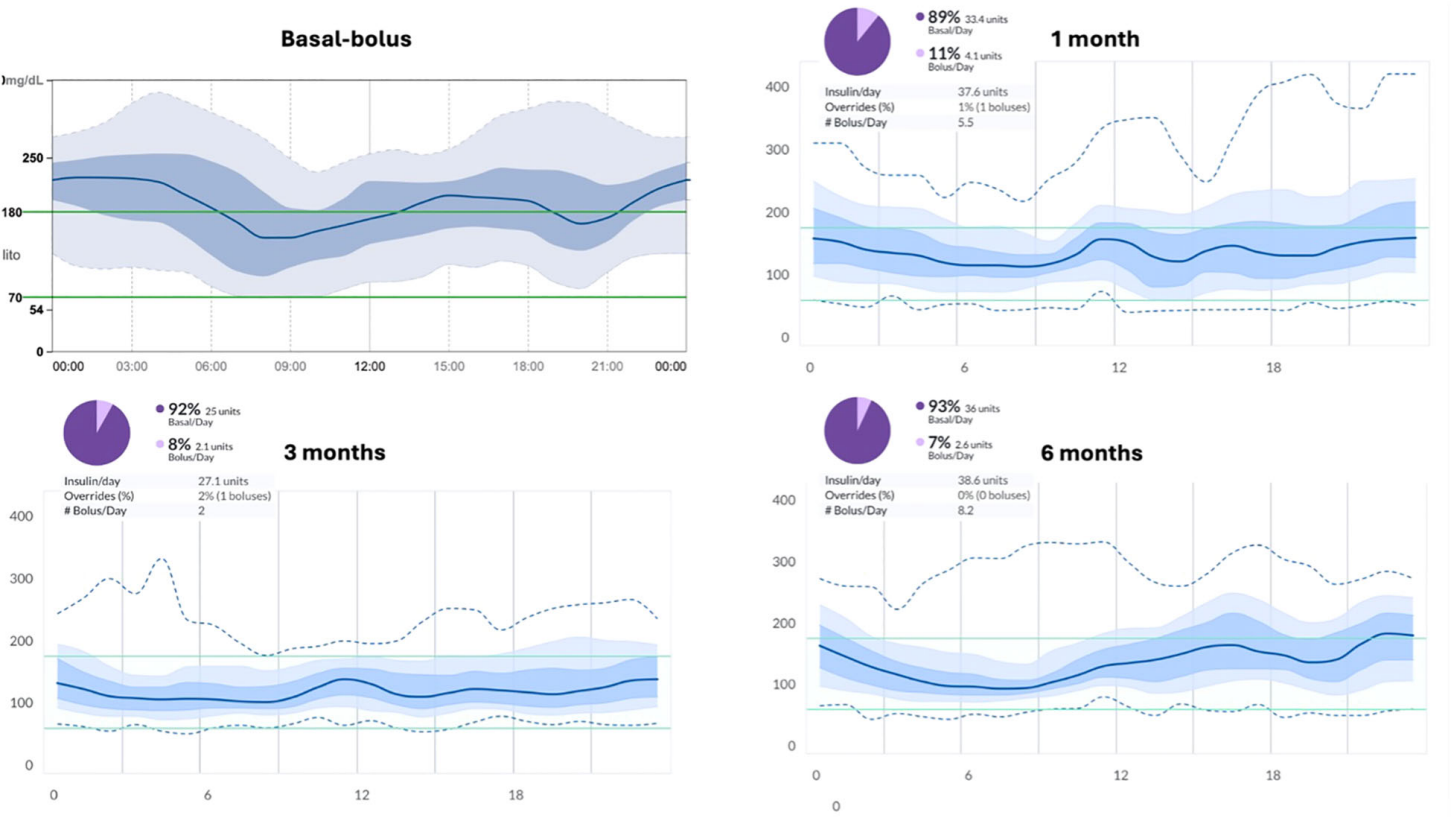
AGP curves showing glucose trends at different timepoints, along with reports of total daily insulin requirements, percentages of basal and bolus insulin administered, and number of boluses delivered.

**Figure 3:**
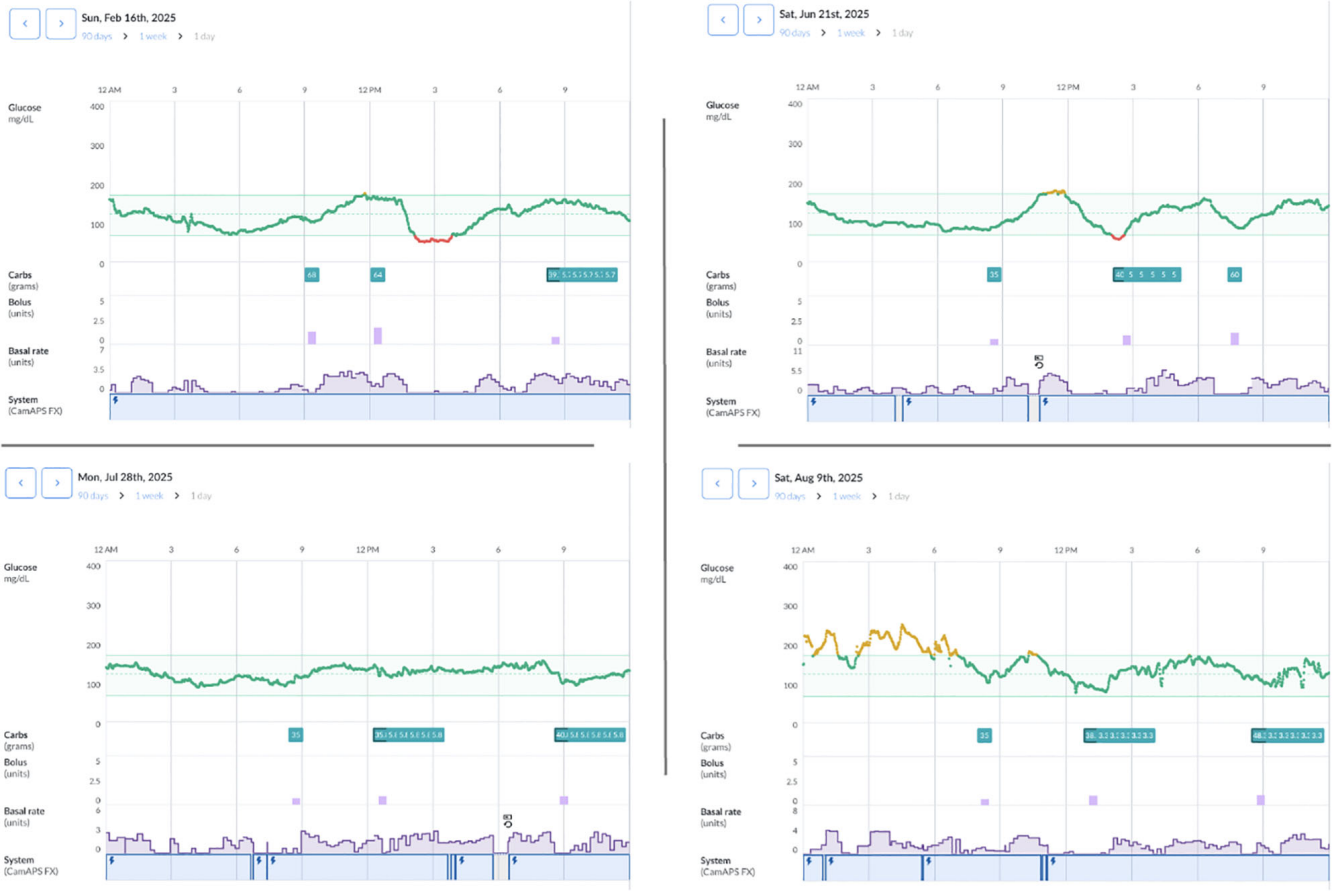
Daily reports illustrating the utilization of the slowly absorbed meal option and its effectiveness in controlling postprandial glucose excursions without inducing hypoglycemia.

After initiation of the mylife Loop, glycemic control improved significantly. Metrics were as follows and showed in **Figure 1**:

- Last 30 days of basal-bolus therapy: TIR 43%, TAR1 40%, TAR2 16%, TBR1 1%, TBR2 0%, CV (Coefficient of variation) 33%, GMI (Glucose management indicator) 7.9%, GRI (Glycemia risk index) 60;
- First 30 days after HCL initiation: TIR 72%, TITR (Time in tight range 70–140 mg/dl) 47%, TAR1 21%, TAR2 4%, TBR1 3%, TBR2 0%, CV 36%, GMI 6.9%, GRI 30;
- At 3 months: TIR 88%, TITR 63%, TAR1 10%, TAR2 1%, TBR1 1%, TBR2 0%, CV 27%, GMI 6.5%, GRI 12;
- At 6 months: TIR 79%, TITR 47%, TAR1 16%, TAR2 3%, TBR1 2%, TBR2 0%, CV 31%, GMI 6.8%, GRI 22;

During follow-up, only minimal adjustments to the insulin regimen were necessary; specifically, the insulin-to-carbohydrate ratio for dinner was slightly modified from 1:45 to 1:40. The improvement in glycemic control is clearly illustrated by the AGP curves shown in **Figure 2**, which compare glucose profiles before and after initiation of the hybrid closed-loop (HCL) system. The patient met the glucose metric goals at all time points, with a substantial increase in TIR and a reduction in GMI. The GRI, a novel composite metric of glycemic quality, also improved significantly. The slow-digestion meal feature was frequently utilized by the patient and proved effective, as illustrated in **Figure 3**. The patient met the glucose metric goals at all time points, with a substantial increase in TIR and a reduction in GMI. The GRI, a novel composite metric of glycemic quality, also improved significantly. Overall, these findings indicate a substantial improvement in the effectiveness of insulin therapy following the introduction of the mylife Loop.

## Discussion

The use of AID systems constitutes a new opportunity to improve glycemic control in patients with pancreatogenic diabetes following acute pancreatitis. This approach may help overcome the unique challenges of insulin management in post-pancreatitis diabetes, where glycemic variability, malabsorption, and impaired counter-regulatory mechanisms complicate conventional insulin therapy. In our case, the adoption of the mylife Loop resulted in a marked and sustained improvement in glucose metrics, with a significant reduction in GMI and an increase in TIR, benefits that persisted at 6 months. To our knowledge, this is the first study reporting the use of the mylife Loop HCL system in post pancreatitis diabetes.

These findings are consistent with the well-established efficacy of Automated Insulin Delivery (AID) systems in type 1 diabetes, where their use has been associated with improved glycemic stability, reduced hypoglycemia, and enhanced quality of life (10, 12). However, despite these promising results, it is important to recognize that the response to advanced diabetes technologies may vary among individuals. Our study suggests that a hybrid closed-loop system may represent an appropriate therapeutic option for post-pancreatitis diabetes, as it enabled meaningful improvements in glycemic control, achieving outcomes that were even superior to those reported with advanced hybrid closed-loop systems in previous studies (13, 14). Given the absence of specific clinical practice guidelines and the lack of randomized controlled trials in post-pancreatitis diabetes, further studies are warranted to confirm these preliminary findings and to define the optimal use of AID systems in this distinctive and complex form of diabetes.

From the patient’s perspective, the transition to mylife Loop was associated with a clear improvement in quality of life. The patient reported a substantial reduction in daily stress related to glucose management, as maintaining stable glycemic levels became easier and largely automated. This sense of control and freedom, together with reduced glycemic fluctuations, contributed to a greater overall well-being and treatment satisfaction.

In conclusion, AID systems may—and should—be considered a valuable therapeutic option for patients with post pancreatitis diabetes who experience unstable glycemic control and marked variability not adequately managed by multiple daily injections or combination therapy. Their use represents an important step toward personalized and automated diabetes management in this challenging condition. Choosing the most appropriate option for each patient and diabetes subtype is challenging; in our experience, the mylife Loop has proven to be particularly suitable for post-pancreatitis diabetes.

## Data Availability

The raw data supporting the conclusions of this article will be made available by the authors, without undue reservation.
